# Bacterial diversity in different regions of gastrointestinal tract of Giant African Snail (*Achatina fulica*)

**DOI:** 10.1002/mbo3.38

**Published:** 2012-10-19

**Authors:** Kiran D Pawar, Sunil Banskar, Shailendra D Rane, Shakti S Charan, Girish J Kulkarni, Shailesh S Sawant, Hemant V Ghate, Milind S Patole, Yogesh S Shouche

**Affiliations:** 1 Molecular Biology Unit, National Centre for Cell ScienceGaneshkhind, Pune, Maharashtra, India; 2 Department of Zoology, Modern College of Arts, Science and CommerceShivajinagar, Pune, 411004, Maharashtra, India; 3 Microbial Culture Collection, Hindustan Antibiotics Ltd. ComplexPimpri, Pune, 411018, Maharashtra, India

**Keywords:** 16S rRNA, bacterial diversity, Giant African Snail, quantitative PCR, T-RFLP

## Abstract

The gastrointestinal (GI) tract of invasive land snail *Achatina fulica* is known to harbor metabolically active bacterial communities. In this study, we assessed the bacterial diversity in the different regions of GI tract of Giant African snail, *A. fulica* by culture-independent and culture-dependent methods. Five 16S rRNA gene libraries from different regions of GI tract of active snails indicated that sequences affiliated to phylum *γ*-*Proteobacteria* dominated the esophagus, crop, intestine, and rectum libraries, whereas sequences affiliated to *Tenericutes* dominated the stomach library. On phylogenetic analysis, 30, 27, 9, 27, and 25 operational taxonomic units (OTUs) from esophagus, crop, stomach, intestine, and rectum libraries were identified, respectively. Estimations of the total bacterial diversity covered along with environmental cluster analysis showed highest bacterial diversity in the esophagus and lowest in the stomach. Thirty-three distinct bacterial isolates were obtained, which belonged to 12 genera of two major bacterial phyla namely *γ-Proteobacteria* and *Firmicutes*. Among these, *Lactococcus lactis* and *Kurthia gibsonii* were the dominant bacteria present in all GI tract regions. Quantitative real-time polymerase chain reaction (qPCR) analysis indicated significant differences in bacterial load in different GI tract regions of active and estivating snails. The difference in the bacterial load between the intestines of active and estivating snail was maximum. Principal component analysis (PCA) of terminal restriction fragment length polymorphism suggested that bacterial community structure changes only in intestine when snail enters estivation state.

## Introduction

Terrestrial gastropods such as the Giant African Snail, *Achatina* (*Lissachatina*) *fulica* Bowdich, 1822, initially originated in East Africa (Bequaert 1950; Simberloff 1995) and are presently distributed across different parts of the world such as Japan (Koyano et al. 1989), the Indian Islands, Australia, Southeast Asia (Shah 1992; Grarff-Teixeira et al. 1995; AFFA 2001; Paiva 2001), and the American continent (Godan 1983). In general, this snail is most abundantly found in areas with high human density. Human presence and activities are the most important factors for the establishment and dispersion of *A. fulica* (De Winter 1989). The inedible parts of this snail are used in animal feed preparation. Besides this economic importance of Giant African Snail, it is generally regarded as a herbivorous animal, feeding primarily on vascular plants (Rauth and Barker 2002) and plant materials with high protein and calcium contents (Iglesias and Castillejo 1994; Chevalier et al. 2003). This snail also participate, with other soil invertebrates, in the decomposition of leaf litter (Hatziioannou et al. 1994) and is considered one of the most destructive pests affecting subtropical and tropical areas, causing large damages to farms, commercial plantations, and domestic gardens. It can also be found on trees, decaying materials in decomposition, and next to garbage deposits (Mead 1995; Vasconcellos and Pile 2001).

Previous studies (Charrier and Rouland 1992; Flari and Charrier 1992) have demonstrated that native cellulose, laminaran, and mannan are highly degraded by snails. Consequently, a large set of bacteria that produce polysaccharide depolymerases and glycoside hydrolases for the digestion of plant materials are involved. The dependence of pulmonates on microbial activity within their gastrointestinal (GI) tract would explain their extraordinary efficiency in plant fiber digestion (60–80%) (Davidson 1976; Charrier and Daguzan 1980). It has been well established that two main features such as (1) an enlarged GI tract region (e.g., crop, intestine, stomach, esophagus, and rectum) and (2) their microbiota unify all animals like *A. fulica*, which feeds on mainly lignocellulosic components of the plant (Dehority 1996; Cazemier et al. 1997; Brune 2003). These two features help *A. fulica* in digestion of lignocellulosic components of the plant. GI tract microbiota plays a very important role, as it provides the host with a battery of digestive enzymes to hydrolyze the plant food. In contrast, the Giant African Snail is reported to harbor potentially pathogenic bacteria in its GI tract and different organs. Akinboade et al. (1980) and Akpavie et al. (2000) showed that various bacteria that are potential pathogens inhabit different organs and tissues including lungs, hemocyanin, liver, kidney, crop, and stomach of clinically healthy Giant African land snails.

Physiologically snails undergo estivation with the advent of adverse conditions. It was reported that the type of plant foods, the length of starvation, the degree of temperature, and percentage of relative humidity are the factors that induce estivation. In estivation state, snails overcome the unfavorable conditions by adjusting their physiological conditions (Rehman and Raut 2010). To our knowledge, a detail survey of culturable and uncultured bacteria in the whole GI tract of this snail has not been carried out so far. Such a survey of the bacterial communities in the whole GI tract of Giant African Snail is expected to shed light on the identity of bacterial population, their abundance (at species, genus, and phylum level), and their role in digestion. Studying the differences in bacterial load and diversity by using molecular methods in active and estivation state will reveal how bacterial load and community structure change when snail enters the latter.

The objectives of this study were to study the abundance and diversity of culturable and uncultured bacteria in the GI tract regions such as esophagus, crop, stomach, intestine, and rectum, and to study how bacterial load and diversity in these GI tract regions change when snail enters estivation state. In this study, the method based on 16S rRNA gene sequencing (culture-independent method) was used to study the diversity and composition of uncultured bacteria in different GI tract regions of the snail. Culture-dependent approach was used to grow and enumerate culturable bacteria from these GI tract regions on Luria Bertani (LB) agar medium. The bacterial load and abundance in each of these regions of active and estivating snails were studied by quantitative real-time polymerase chain reaction (qPCR). Culture-independent molec-ular method terminal restriction fragment length polymorphism (T-RFLP) was used to study the differences in bacterial diversity among the GI tract regions of active (*n* = 5) and estivating (*n* = 5) snails.

## Materials and Methods

### Sampling and dissection of snails

For culture-dependent and culture-independent study, snails (*n* = 3) in active state were collected from different locations around Pune, Maharashtra, India and immediately processed. For qPCR and T-RFLP analysis, snails in active (*n* = 5) and estivation (*n* = 5) state were collected. Animals were refrigerated for 30 min at 5°C for immobilization, surface sterilized with 70% ethanol (v/v) and their shells were carefully broken to expose soft body. Animals were then aseptically dissected to separate whole GI tract. From the whole GI tract, esophagus, crop, stomach, intestine, and rectum were separated and used.

### DNA extraction and PCR amplification

DNA from each GI tract region under study was extracted by using DNeasy Blood Tissue Kit (Qiagen, U.K.) following the manufacturer's instructions. DNAs from each GI tract regions of three snails were isolated, solvated in double distilled water, and then quantified on Nanodrop ND1000 spectrophotometer (Nanodrop Technologies, Wilmington, Delaware). For construction of 16S rRNA gene library, each of the three DNAs from each GI tract region was used separately in PCR reaction. PCR was performed with the bacteria-specific primer pair 27F (5′-AGAGTTTGATYMTGGCTCAG) and 907R (5′-CCGTCAATTCMTTTGAGTTT) (Weisburg et al. 1991). The 50 μL PCR reaction consisted of 4 μL (10 ng/μL) of template DNA, 5 μL of 10× buffer (Bangalore Genei, India), 0.2 mmol/L dNTPs (Bangalore Genei, India), 10 pmol of each of primers, and 0.5 U of *Taq* polymerase (Bangalore Genei, India). The thermal cycling conditions were set with the following program: an initial 3-min denaturation at 95°C, 30 cycles of denaturation (1 min at 94°C), annealing (1 min at 55°C) and extension (1 min at 72°C), and a final 7-min extension at 72°C. All PCRs were carried out in triplicates and products were checked for size and purity on 1% (w/v) agarose gels.

### 16S rRNA gene library construction, sequencing, and phylogenetic analysis

The PCR products were purified using PEG–NaCl precipitation (Sambrook et al. 1989) and TA cloning was performed using a TOPO TA cloning kit (Invitrogen, Bangalore, India). Two hundred and fifty clones from each library (1250 in total from whole GI tract) were randomly picked, and sequencing was performed using the ABI Big-Dye version 3.1 sequencing kit as per the manufacturer's instructions, with both M13F and M13R primers. The generated sequences were analyzed using ChromasPro software (http://www.technelysium.com.au/ChromasPro.html) and compared with the current database of nucleotide sequences at GenBank and Ribosomal Database Project (RDP). Reference sequences were chosen on the basis of BLASTn similarities. All 16S rRNA gene sequences were checked for possible chimeric artifacts using the Pintail program (Ashelford et al. 2006) in conjunction with Bellerophon (Huber et al. 2004). Multiple sequence alignments of 16S rRNA gene sequences were performed with ClustalW, Version 1.8 (Thompson et al. 1994), and were edited manually using DAMBE (Xia and Xie 2001) to obtain an unambiguous sequence alignment. Nucleotide distance matrices were constructed with DNADIST from PHYLIP version 3.61 (Felsenstein 1989) using the Kimura two-parameter model (Kimura 1980). Operational taxonomic units (OTUs) were generated using the DOTUR program (Schloss and Handelsman 2005) at 97% sequence similarity cutoff. The sequences representing each OTU were compared with NCBI database using BLASTN, and the closest matches to bacterial strains were obtained. A Bayesian method was used for the construction of phylogenetic trees. Before Bayesian inference analysis, a DNA substitution model for the complete data set was selected using MrModeltest2 (Posada and Crandall 1998) and the Akaike Information Criterion (AIC). The phylogenetic trees were constructed using MrBayes, version 3.0b4 (Ronquist and Huelsenbeck 2003). Abundance-based coverage estimator (ACE) estimate, ACE coverarge %, Chao1 richness estimates, Chao1 richness %, and Simpson Index of diversity were calculated, and rarefaction curves were constructed by DOTUR to assess microbial diversity richness.

### UniFrac analysis

For weighted UniFrac distance metric analyses, the sequences were aligned with Clustal W and a phylogenetic tree was constructed using MrBayes. The phylogenetic tree was exported in Newick format and the environmental file linking the sequences to different GI tract regions was used in the UniFrac calculations. As a result, Unweighted Pair-Group Method with Arithmatic Mean (UPGMA, a technique that merges the closest pair of environments or cluster of environments at each step) cluster of samples based on phylogenetic lineages they contained was created.

### Enumeration, isolation, and sequencing of culturable bacteria

Culturable bacteria were enumerated and isolated from each GI tract region of the active snail. Weighed quantity of tissue of each GI tract region was placed in 1 mL of phosphate buffered saline (PBS) in 1.5 mL microcentrifuge tube, homogenized using sterile plastic homogenizer and was serially diluted through 10^−10^ dilutions. Twenty microliters of each dilution was plated on LB Agar medium and then incubated at 37°C for 48 h. Colonies were classified based on morphological parameters of shape, color, margins, elevation, and texture. A representative of each colony morphology was purified by subculturing. Extraction of total genomic DNA, amplification of 16S rRNA gene, and sequencing were performed as described previously (Pidiyar et al. 2004). The sequences representing each bacterial isolate were compared with NCBI database using BLASTN, and the closest matches to bacterial isolates were retrieved.

### qPCR analysis

*TaqMan* probe-based qPCR analysis was performed to quantify total bacterial load in each GI tract region of active and estivating snails. Total 16S rRNA gene copy numbers in each GI tract region were estimated in physiologically active (*n* = 5) and estivating snails (*n* = 5). The qPCR analyses were performed with the 7300 Real-Time PCR System (Applied Biosystems, Carlsbad, California) using 96-well optical-grade PCR plates (Axygen, India) covered with optical-quality sealing film (Bio-Rad, USA). Each reaction was carried out in triplicate by using *TaqMan* Universal PCR Master Mix (Applied Bioscience), 331F and 797R primers (numbering based on *Escherichia coli* 16S rRNA gene) (Doung-udomdacha et al. 2000) and (6-FAM)-5′-CGTATTACCGCGGCTGCTGGCAC-3′-(TAMRA) probe. Purified *Lactococcus lactis* DNA was used in 1:10 dilution series (10 pg–100 ng) as a standard. Each 25 μL reaction consisted of 12.5 μL of *TaqMan* Universal Master Mix, 600 nmol/L of each of forward and reverse primers (Sigma-Aldrich, USA), 100 nmol/L of probe (Sigma-Aldrich, Bangalore, India), and 800 pg of template DNA. The thermal cycling conditions were 50°C for 2 min and 95°C for 5 min followed by 40 cycles of denaturing at 95°C for 15 sec, primer annealing at 60°C for 1 min, and DNA extension at 72°C for 1.5 min. The C_T_ values were determined on the basis of the fluorescence signals at the mean baseline during the early cycles of amplification. PCR efficiency was calculated by using following equation: Efficiency = 10 (−1/slope) − 1.

### T-RFLP analysis

For T-RFLP analysis, the same DNA samples extracted for qPCR analysis were used. Partial 16S rRNA gene was amplified for T-RFLP analysis using bacterial 16S primers 27F labeled with carboxyfluorescein (6-FAM) and unlabeled 907R. The 50 μL PCR reaction consisted of 4 μL (10 ng/μL) of template DNA, 5 μL of 10× buffer (Bangalore Genei, India), 0.2 mmol/L dNTPs (Bangalore Genei, India), 10 pmol of each of primers, and 0.5 U of *Taq* polymerase (Bangalore Genei, India). The thermal cycling conditions were set with the following program: an initial 3-min denaturation at 95°C, 30 cycles of denaturation for 1 min at 94°C, annealing for 1 min at 55.6°C, extension for 1 min at 72°C, and a final 7-min extension at 72°C. The PCR reactions were carried out in triplicate for each sample, resulting products were pooled, purified using a PCR purification kit (Sigma-Aldrich, USA) and quantified on Nanodrop ND1000 spectrophotometer. Hundred nanograms of purified PCR products was digested separately by using five units of restriction endonucleases Hind III and Bgl II (New England Biolabs, UK) in a final reaction volume of 20 μL for 3 h. The 10 μL aliquots of digested products were precipitated with 0.1 v/v 3 mol/L sodium acetate; pH 4.8 at 25°C and two volumes of absolute ethanol. Precipitate was pelleted down by centrifugation at 15,000 rpm for 10 min and washed twice with 70% ethanol (v/v). The washed pellet was carefully dissolved in 9.5 μL of HiDi Formamide (Applied Biosystems, USA) and 0.5 μL Gene Scan 500 LIZ size standard (Applied Biosystems, USA). Samples were denatured by heating at 95°C for 5 min, immediately placed on ice and then loaded onto ABI 3730xl DNA analyzer (Applied Biosystems, USA). Restriction fragments including the detectable terminally labeled restriction fragments were size-separated and compared with internal lane molecular size standard by the GeneScan mode of the sequencer. Fragment separation data were collected, analyzed with GeneMapper analysis software (version 4.0), and used to generate output data in binary matrix. Data in binary matrix were used for principal component analysis (PCA). PCA was performed on NTSYS PC version 2.1 (Ralf 2000).

### Nucleotide sequence accession numbers

All the clone library sequences generated from this study have been deposited at the NCBI GenBank database with the following accession numbers – rectum: JN211197–JN211221; crop: JN211222–JN211248; esophagus: JN211249–JN211278; intestine: JN211279–JN211305; and stomach: JN211306–JN211314. The accession numbers for sequences from cultured isolates are as follows: crop: JN251751–JN251757; intestine: JN251758–JN251762; esophagus: JN251763–JN251769; rectum: JN251770–JN251777; and stomach: JN251778–JN251783.

## Results

### Phylum-level distribution of bacteria in clone libraries

**Figure 1:**
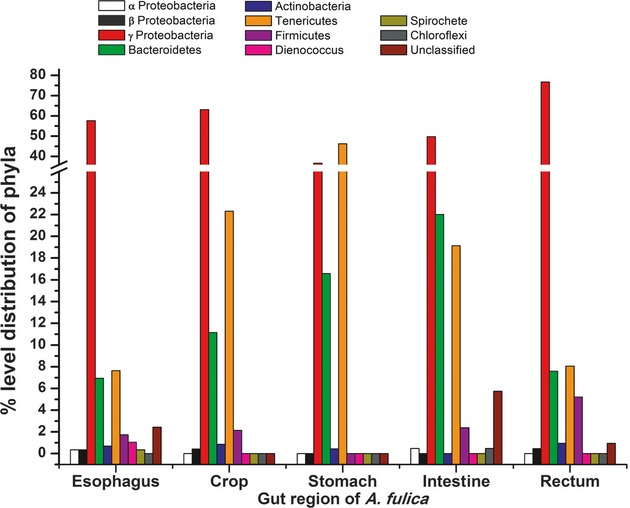
Composition of 16S rRNA gene clone libraries. Phylum-level classification of bacterial phylotypes in the different GI tract regions of active *Achatina fulica* (*n* = 3).

A total of 228, 233, 229, 209, and 211 chimera-free sequences (1110 in total) were obtained and analyzed from esophagus, crop, stomach, intestine, and rectum, respectively, in this study. The sequences in libraries from all GI tract regions could be mapped to 10 bacterial phyla namely *α-Proteobacteria*, *β-Proteobacteria*, *γ-Proteobacteria*, *Bacteroidetes*, *Actinobacteria*, *Tenericutes*, *Firmicutes*, *Deinococcus–Thermus*, *Spirochetes*, and *Choloroflexi* (Fig. 1). Sequences phylogenetically affiliated to phylum *γ-Proteobacteria* dominated esophagus (57.63%), crop (63%), intestine (49.76%), and rectum (76.77%) libraries, whereas in stomach, *Tenericutes* (46.28%) was the dominant phylum followed by *γ-Proteobacteria* (36.68%). Seven percent, 22%, 19%, and 8% sequences of esophagus, crop, intestine, and rectum, respectively, were phylogenetically affiliated to *Tenericutes*, whereas 1.7% sequences of esophagus, 2.1% sequences of crop, 2.3% sequences of intestine, and 5.2% sequences of intestine libraries were related to *Firmicutes*. Very few (<1%) sequences in esophagus, crop, stomach, and rectum libraries were related to *Actinobacteria*, but in intestine library, they were completely absent. *Bacteroidetes* was the third most abundant phylum in all libraries with 7%, 11%, 16%, 22%, and 7% sequences in esophagus, crop, stomach, intestine, and rectum libraries, respectively. Maximum bacterial diversity was observed in esophagus since sequences phylogenetically related to *Deinococcus–Thermus* (1%), *Spirochetes* (0.3%), *α-Proteobacteria* (0.3%), *β-Proteobacteria* (0.3%), and unclassified bacteria (2.4%) were also retrieved. In crop and stomach libraries, sequences affiliated to *Deinococcus–Thermus*, *Spirochetes*, *α-Proteobacteria*, and unclassified bacteria were not observed. Similarly, in rectum and intestine libraries, members of *Deinococcus–Thermus* and *Spirochetes* were not observed.

### Comparison and estimation of bacterial diversity in clone libraries

**Figure 2:**
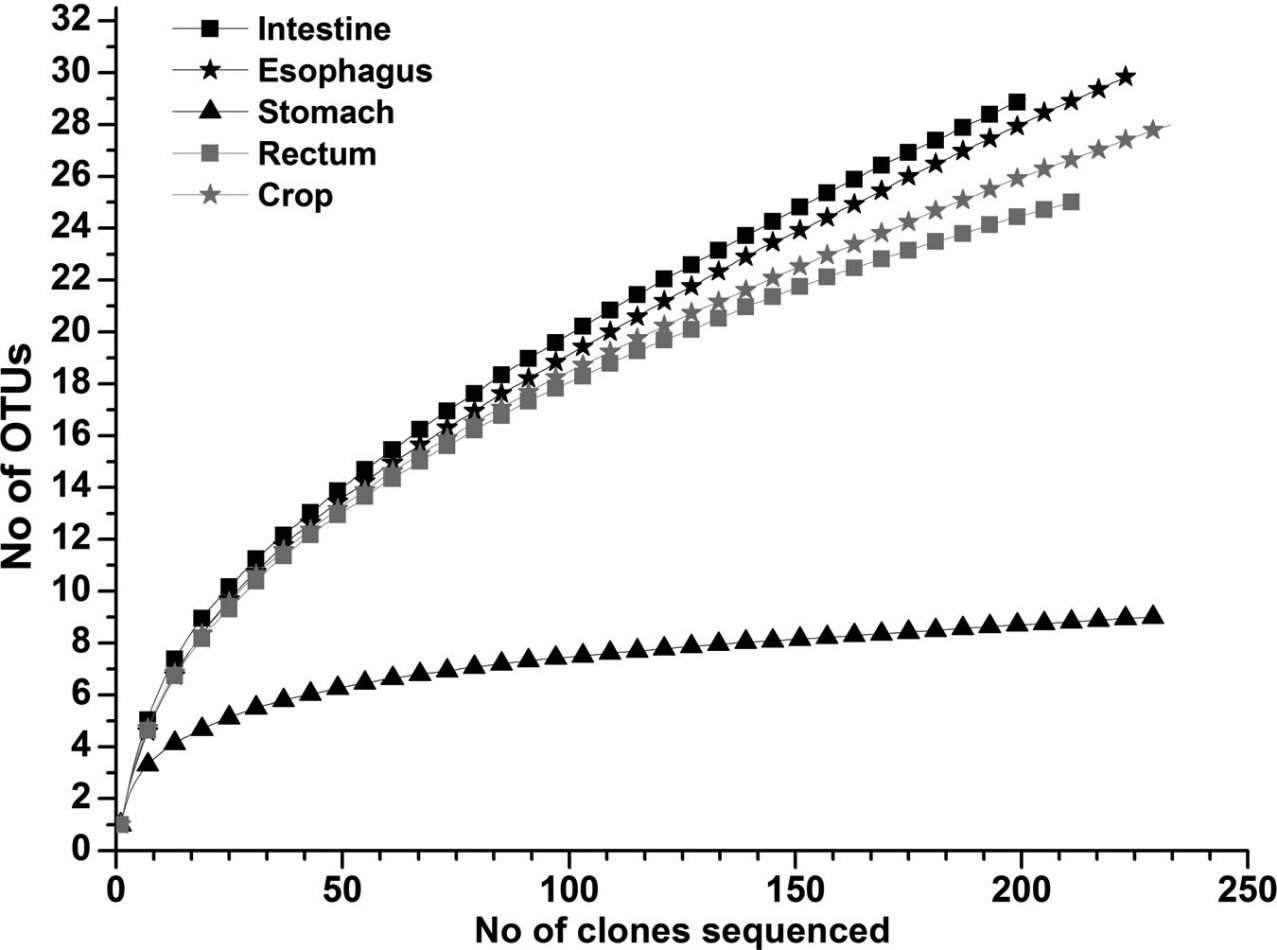
Rarefaction curves generated for 16S rRNA gene clone libraries from the different GI tract regions of *Achatina fulica*.

In phylogenetic analysis, representative OTU from individual OTU groups was defined using a 3% sequence identity cutoff value by the furthest-neighbor module of DOTUR. DOTUR analysis of chimera-free sequences from each GI tract region identified 30, 27, 9, 27, and 25 OTUs (118 in total) from esophagus, crop, stomach, intestine, and rectum libraries, respectively. The rarefaction curves for all libraries except stomach were similar suggesting that the number of clones sequenced were insufficient to reach saturation and indicated that further sequencing would reveal additional unique OTUs (Fig. 2). In the case of stomach library, sequencing 229 clones was sufficient to reach saturation (Fig. 2). In the esophagus library, the most abundant OTU (SN_OE 68), which constituted 36.8% of the total number of sequences, was affiliated to *Citrobacter freundii* and the second most abundant OTUs (SN_OE 197 and SN_OE 217) that constituted 10.9% of the total number of sequences were affiliated to *Kluyvera ascorbata* and *Mycoplasma gypis*, respectively (Fig. S1).

In the crop library, the most abundant OTU (SN_CP 54), which constituted 25.7% of the total number of sequences, was affiliated to *C. freundii*. Interestingly, the second most abundant OTU (SN_CP 2) that constituted 22.7% of the total number of sequences was affiliated to an uncultured bacterium (EU637704)*. Mycoplasma*-related OTUs (SN_CP 29 and SN_CP 44) were represented by 12.8% and 9.4% of the total number of clones, respectively (Fig. S2). In contrast to the esophagus and crop libraries, the stomach library was dominated by *Mycoplasma*-related clones. The most abundant OTU (SN_ST 4) affiliated to *Mycoplasma* sp. was represented by 46% of the clones, while OTU (SN_ST 6) affiliated to *K. ascorbata* accounted for 24% of clones. The third most abundant OTU (SN_ST 183) in the stomach library was affiliated to *Pedobacter* sp. (16.5% of the clones) (Fig. S3).

Two OTUs affiliated to *C. freundii* (SN_IN 13) and *Enterobacter amnigenus* (SN_IN 20) were the most abundant in the intestine library, and each was represented by 19% sequence abundance. Similar to crop library, the second most abundant OTU (SN_IN 85) was affiliated to an uncultured bacterium (FJ228907) (12% of the clones)*. Pedobacter rhizospharae*-related OTU (SN_IN 9) was the third most dominant in intestine library that accounted for 9% of the total number of sequences (Fig. S4). The rectum library was exclusively dominated by *C. freundii-*related clones. The two most dominant OTUs (SN_REC 124 and SN_REC 2) representing 27.9% and 23.6% of the total number of sequences, respectively, were affiliated to *C. freundii*. OTUs affiliated to *K. georgiana* (SN_REC 28) and uncultured bacterium (SN_REC 14) were the third most abundant, and each was represented by 9% of the total number of sequences (Fig. S5).

Seven OTUs were shared between esophagus and crop followed by four OTUs between crop and rectum, three OTUs between crop and stomach, and two OTUs between esophagus and stomach. Only one OTU each was shared between stomach and intestine, intestine and esophagus, rectum and esophagus, crop and intestine while not a single OTU was shared among all the libraries (Fig. S6). Of the 118 OTUs observed in this study, only three OTUs were shared by more than two libraries. OTU phylogenetically affiliated to *C. freundii* (AB548829) was shared by esophagus, crop, and intestine; OTU affiliated to *K. georgiana* (AM933755) was shared by esophagus, crop, and rectum, whereas OTU affiliated to *K. ascorbata* (FN813249) was shared by esophagus, crop, and stomach.

Estimations of the fraction of total bacterial diversity covered in whole GI tract ranged from 28.16% (esophagus) to 94.73% (stomach), depending on the estimation model used. The true diversity of different samples estimated by the ACE model ranged from 11 (stomach) to 87 (esophagus) species (coverage: 34–82%), and with the Chao model from 9 to 106 species (coverage: 28–94%). Simpson's index of diversity ranged from 0.12 (intestine) to 0.3 (stomach) while Shanon's index ranged from 1.4 (stomach) to 2.4 (intestine) (Table 1). The highest diversity was found in the esophagus and lowest in the stomach.

**Table 1:** Estimation of bacterial diversity in the different GI tract regions of *Achatina fulica*

GI tract region	No. of sequences	No. of OTUs	ACE estimate	ACE coverage %	Chao1 estimate	Chao1 coverage %	Simpson's Index	Shannon's index
Esophagus	228	30	87.18	34.41	106.5	28.16	0.17	2.27
Crop	233	28	57.33	48.84	46.20	60.60	0.14	2.32
Stomach	229	9	10.91	82.49	9.5	94.73	0.30	1.42
Intestine	209	27	66.41	40.65	59	45.76	0.12	2.45
Rectum	211	25	41.58	60.12	32.5	76.92	0.15	2.26

The table shows number of phylotypes observed and estimated species richness, coverage, and diversity indices for 16S rRNA clone libraries from the different GI tract regions. Numbers were calculated with the DOTUR program; OTUs were defined using a distance level of 3%.

UniFrac cluster analysis was used to compare the bacterial communities on the basis of phylogenetic information. In the cluster analysis of the clone libraries, bacterial community in stomach was well separated from the cluster formed by other GI tract regions (Fig. S7). This was in consistence with the low bacterial diversity observed in stomach.

### Diversity of cultured bacteria

By culture-dependent method, total viable counts (TVC) in each GI tract region under study was enumerated, and it was observed that TVC in whole GI tract ranged from 10^3^ to 10^6^ (data not shown). Thirty-two, 35, 19, 32, and 23 isolates (134 in total) from esophagus, crop, stomach, intestine, and rectum, respectively, were screened. Of these 134 isolates screened initially, 33 distinct isolates were obtained from all GI tract regions under study (Table 2). These isolates mainly belonged to two main bacterial phyla namely *γ-Proteobacteria* and *Firmicutes*. Based on the 16S rRNA gene sequence, these 33 isolates showed their closest matches to 6 distinct genera of each of *γ-Proteobacteria* (*Citrobacter*, *Kluyvera*, *Actinobacteria*, *Escherichia*, *Shigella*, and *Salmonella*), *Firmicutes* (*Staphylococcus*, *Bacillus*, *Enterococcus*, *Lactococcus*, *Kurthia*, and *Exiguobacterium*), and one of *β-Proteobacteria* (*Acidovorax*) (Table 2). In esophagus and crop, members of the genera *Lactococcus* and *Staphylococcus* were the most predominant which accounted for 50% and 77% of total numbers of isolates identified, respectively. Likewise, members of genus *Lactococcus* were also dominant in stomach, intestine, and rectum accounting for 42%, 68%, and 34.7% of the isolates identified, respectively. Among the members of *Lactococcus*, *L. lactis* was the most dominant and common to esophagus, stomach, intestine, and rectum, while in crop, *Staphylococcus saprophyticus* was the dominant member. Species of *Shigella* (*S. boydii)* and *Exiguobacterium* (*E. acetylicum*) were observed only in rectum, whereas species of *Salmonella* were observed only in the stomach (Table 2).

**Table 2:** Abundance of bacterial isolates within the bacterial phylum and genus

Phylum	Genus	Esophagus	Crop	Stomach	Intestine	Rectum
*γ*-*Proteobacteria*	*Citrobacter*	9 (28)	2 (5.7)	5 (26)	–	6 (26)
	*Kluyvera*	–	1 (2.8)	–	3 (12)	–
	*Acinetobacter*	2 (6.2)	–	1 (5.6)	–	–
	*Escherichia*	2 (6.2)	–	–	–	–
	*Shigella*	–	–		–	2 (8.6)
	*Salmonella*	–	–	1 (5.6)	–	–
*β*-*Proteobacteria*	*Acidovorax*	1 (3.1)	–	–	–	–
*Firmicutes*	*Staphylococcus*	–	27 (77)	–	2 (8)	–
	*Bacillus*	–	2 (5.7)	–	–	1 (4)
	*Enterococcus*	–	1 (2.8)	–	–	1 (4.3)
	*Lactococcus*	16 (50)	1 (2.8)	8 (42)	17 (68)	8 (34.7)
	*Kurthia*	2 (6.2)	1 (2.8)	4 (21)	3 (12)	4 (17)
	*Exiguobacterium*	–	–	–	–	1 (4.3)

Distribution of bacterial isolates and their abundance in the different GI tract regions. Values are number of isolates identified; values in parentheses correspond percentage of the total number of isolates identified.

### Analysis of bacterial load by qPCR

**Figure 3:**
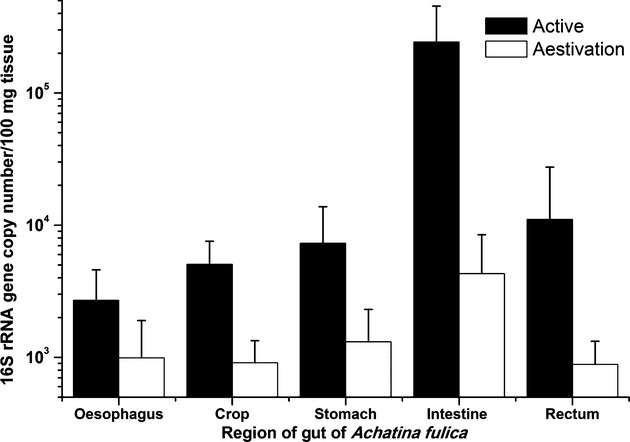
qPCR analysis of total bacterial load in the different GI tract regions of *Achatina fulica*. Figures are mean of results from three replicates. Bars denote standard deviation values.

*TaqMan* probe-based qPCR analysis was performed to study the differences in bacterial load in the different GI tract regions of active and estivating snails. qPCR analysis was performed to quantify 16S rRNA gene copy numbers in all GI tract regions of active (*n* = 5) and estivating snails (*n* = 5). Standard curve was prepared using genomic DNA from *L. lactis*. DNA standard for 16S rRNA gene quantification showed a slope of −3.15 which corresponds to about 107% PCR efficiency. qPCR-based quantification of total bacteria showed a significant difference in bacterial load between different GI tract regions under study (Fig. 3). In the whole GI tract of active snail, 16S rRNA gene copy numbers ranged from 2.6 × 10^3^ (100 mg tissue)^−1^ (esophagus) to 2.4 × 10^5^ (100 mg tissue)^−1^ (intestine), whereas in whole GI tract of snail in estivation state, it ranged from 8 × 10^2^ (100 mg tissue)^−1^ (rectum) to 4.3 × 10^3^ (100 mg tissue)^−1^ (intestine). Thus, in active state, highest bacterial load was observed in intestine and lowest in esophagus, whereas in estivation state, lowest bacterial population was noted in rectum and highest in intestine. Overall, low bacterial load was observed in estivating snails in comparison with active snails.

### T-RFLP analysis

Furthermore, how bacterial communities in different GI tract regions of snail change when they enter estivation was studied. For this purpose, the culture-independent molecular method T-RFLP was used. For analyzing the bacterial community in whole GI tract, the same DNA samples (extracted for qPCR analysis) from GI tract regions of snails in active (*n* = 5) and estivations state (*n* = 5) were used.

**Figure 4:**
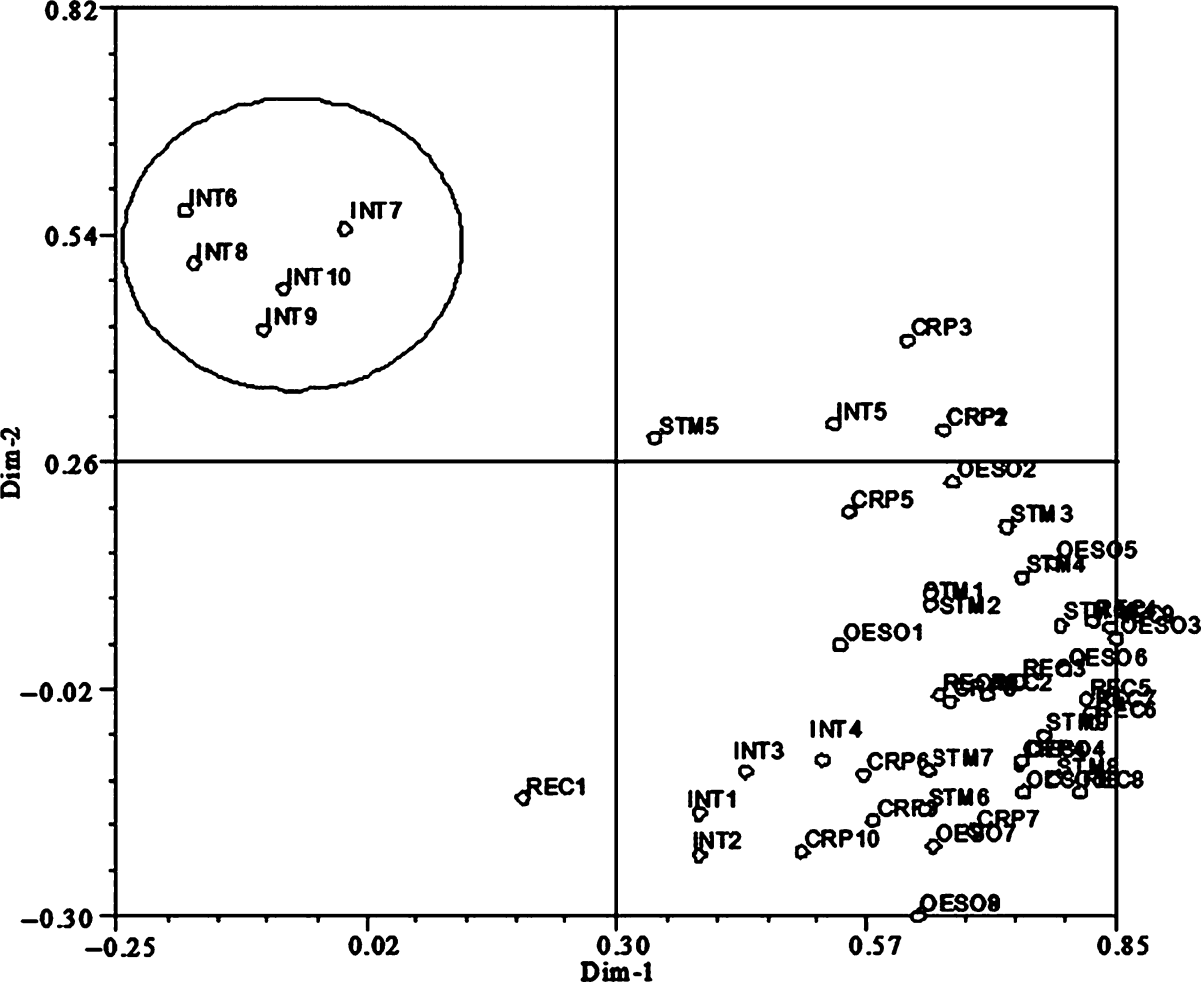
Principal component plot for T-RFLP profile of 16S rRNA genes amplified from the different GI tract region samples from active and estivating snails. OESO, esophagus; CRP, crop; STM, stomach; INT, intestine; REC, rectum. Sample numbers 1–5: samples from active snails. Sample numbers 6–10: samples from estivating snails.

Two replicate T-RFLP profiles were generated per sample by performing PCR twice on each DNA extract. Each PCR product was used to generate a separate T-RFLP profile for that sample/DNA extract. The area of each terminal restriction fragment (T-RF) that appeared in both replicate profiles was averaged to create a composite profile that was used for analysis. By excluding T-RFs less than 1% of profile area, our analysis may provide a conservative estimate of bacterial diversity. The T-RFs of size between 50 and 850 bp were analyzed and total of 66 and 69 polymorphic T-RFs from *Hind* III and *Bgl*II (135 from both enzymes) restricted samples, respectively, were analyzed. Using GeneMapper analysis software, data were converted into binary matrix and used for PCA on NTSYS. In PCA, intestine samples from estivating snails were clustered together, and all other GI tract region samples from both snail types were clustered together. PCA analysis of the T-RFLP profiles from each GI tract region of both snail types, except intestine of estivating snail, revealed considerable overlap of the structure of the communities. This, with the exception of intestine samples from estivating snails, indicated a high degree of similarity of microbial communities in the different GI tract regions of sampled snail individuals (Fig. 4). The clustering or grouping in PCA suggested that except in intestine of estivating snails, microbial communities in all GI tract regions of active and estivation snail were similar. This trend of clustering in PCA clearly indicated that bacterial community structure only in intestine changes when they enter estivation state.

## Discussion

From the environmental and ecological point of view, the terrestrial gastropod *A. fulica* holds importance because of its herbivorous nature. The bacterial communities in GI tract of this snail may have important role to play in digestion of plant fiber and other material. While initiating this study, we assumed that the different GI tract regions such as the esophagus, crop, stomach, intestine, and rectum are highly specialized compartments, and each could have a distinct role to play in digestion. These functionally specialized GI tract regions may be unique microenvironments and could harbor unusual bacterial communities. Our clone library analyses of different GI tract regions of active *A. fulica* indicated *Proteobacteria* as the most abundant phylum. Previously, the members of *Proteobacteria* were also observed as most dominant in intestines of three other species of Planorbidae (Gastropoda: Pulmonata) such as *Biomphalaria pfeifferi*, *Bulinus africanus,* and *Helisoma duryi* (Van Horn et al. 2011). The results obtained with the ACE model, the Chao model, Simpson's index, and Shanon's index of diversity were fairly congruent with one another. Also, the high diversity in esophagus and low diversity in stomach were in congruence with the estimate of total bacterial diversity drawn from different estimation models used (Fig. 2 and Table 1). The low diversity in stomach is most likely not an artifact of limited sampling efforts as indicated by the rarefaction analyses of clone libraries. The cloning, sequencing, and analysis of the stomach sample were repeated, and similar results were observed indicating low diversity (data not shown). The low diversity in stomach can be attributed to the digestive physiology and pH milieu of the stomach. As reported by Misra and Shrivastava (1994), the pH in stomach of gastropod molluscs is more acidic than other GI tract regions. Moreover, digestion in stomach is carried out by enzymes produced by digestive glands or cecae. The low bacterial diversity in stomach may be as a result of acidic environment and extracellular enzymatic digestion.

As revealed by our results, the conspicuous feature of the bacterial communities in the snail GI tract was the abundance of bacteria affiliated to *Tenericutes*. Among *Tenericutes*, members of *Mycoplasma* and uncultured bacteria were the dominant. *Mycoplasma gypis* was observed in both esophagus and crop libraries, whereas *M. alvi* and *M. spumans* were observed only in esophagus and crop libraries, respectively. Members of *Tenericutes* from intestine and rectum libraries were identified as uncultured bacterium. We assume that ours is the first study demonstrating the putative presence of *Mycoplasma*-related members in *A. fulica*. *Mycoplasmas* are widely distributed, smallest forms of host-specific, commensal bacteria colonizing a wide range of plants, insects, reptiles, birds, mammals, and humans. They are mostly known as avian pathogens of poultry and some passerines with which they cause significant economic losses (Razin et al. 1998). *Mycoplasma* species are pathogenic for their hosts and can, under certain conditions, cause diseases in the hosts (Maniloff et al. 1992; Razin et al. 1998). While our results suggest the presence of *Mycoplasma-*related members in the GI tract of *A. fulica,* further investigations will be needed to ascertain whether they are pathogenic or carry out some specialized function in the GI tract. Another apparent feature of bacterial communities in snail GI tract was the abundance of members of the genus *Citrobacter*, a genus of Gram-negative coliform bacteria in the Enterobacteriaceae family, in the esophagus, crop, intestine, and rectum. The most dominant OTUs in all these GI tract regions were affiliated to *C. freundii. Citrobacter freundii* and few other members of Enterobacteriaceae were linked to human disease such as acute necrotizing pancreatitis (Samonis et al. 2009). Recently, *C. freundii* was also detected in a sample obtained from a patient with pancreatic pseudocyst after an acute necrotizing pancreatitis (Lozano-Leon et al. 2011).

Furthermore, characterization of bacterial communities in GI tract by culture-dependent method identified *γ-Proteobacteria* and *Firmicutes* as major groups. This was in congruence with our clone library data which also found *γ-Proteobacteria* and *Firmicutes* as major groups. In terms of number of genera observed, crop and rectum were the most diverse as bacterial isolates from these two regions belonged to seven genera each. Presence of *L. lactis* throughout the GI tract might be symptomatic of the feeding habit of *A. fulica*. *Lactococcus lactis* is one of the most important nonpathogenic bacteria involved in the dairy industry. This bacterium is widely used for manufacturing dairy products like buttermilk, yogurt, and cheese. Moreover, it is also used to prepare pickled vegetables, beer, wine, some breads and sausages, and other fermented foods. The presence of this bacterium indicates waste food material as a possible source of this bacterium in the GI tract of *A. fulica*. It is interesting to know that members of *Shigella* and *Exiguobacterium* were exclusively present only in rectum. *Exiguobacterium acetylicum* is a Gram-positive, rod-shaped, yellow-pigmented bacterium and has been previously isolated from an apple orchard rhizospheric soil (Selvakumar et al. 2009). Another apparent feature of our culture-dependent study is the presence of *Kurthia gibsonii,* a member of *Firmicutes* in all GI tract regions of snail. Although at relatively low abundance, *K. gibsonii* was consistently present in all GI tract regions. Members of *Kurthia* were previously isolated from intestinal content of chickens feces, water and milk (Charrier et al. 2006). Our study is the first report indicating the presence of *K. gibsonii* in the GI tract of *A. fulica*. The presence of *L. lactis*, *K. gibsonii*, and *E. acetylicum* is symptomatic of the feeding habit and habitat of *A. fulica*. Members of *Lactococcus*, *Kluyvera*, *Shigella*, *Staphylococcus*, *Bacillus*, and *Enterococcus* were observed by both culture-dependent and culture-independent methods. To our knowledge, no studies previously attempted to isolate bacteria from the GI tract of *A. fulica*. Charrier and colleagues (2006) had previously isolated cultivable fermentative bacteria from the intestines of two edible snails, *Helix pomatia* and *Cornu aspersum*. Similar to the GI tract of *A. fulica,* bacterial isolates from these two edible snails belonged to *γ-Proteobacteria* and *Firmicutes*. Our study is unique in that culture-dependent and culture-independent methods were compared directly for the qualitative assessment of bacterial communities in different GI tract regions.

In which region of the GI tract, bacterial load changes when snail enters estivation state was further investigated. For the purpose of this study, snails in active and estivation state were identified based on the absence or presence of heavily calcified plate known as Pomum or Epiphragm. The total bacterial population in each GI tract region was quantified using *TaqMan* probe-based PCR method. Total bacteria were determined using universal primers that hybridized to all bacteria. For an accurate estimation of total number of bacteria, DNA standard should be constructed with the DNA from the bacteria that predominate the given habitat. In order to minimize the variations caused by differences in 16S rRNA gene copy number and effect of generation time of the bacteria during estimation, a purified DNA of *L. lactis*, a dominant bacterium among cultured isolates, was used for generating standard graph. qPCR-based quantification of 16S rRNA gene clearly indicated that bacterial load in all GI tract regions decreases when *A. fulica* undergoes estivation. The total viable counts from each of the GI tract region were fairly in congruence with the results obtained by qPCR. When snails enter estivation, 56 times decrease in bacterial load was observed in intestine followed by 12.5, 5.5, 5.4, and 2.7 times decrease in rectum, stomach, crop, and esophagus, respectively.

In order to ensure restriction enzymes discriminate the different groups in snail GI tract, an analytical web server REPK (http://rocaplab.ocean.washington.edu/tools/repk) (Collins and Rocap 2007) was used to select restriction endonucleases: *Hind*III and *Bgl*II. OTU sequences from 16S rRNA clone library analyses were used as input data in REPK to select these restriction enzymes. qPCR analysis and the clustering obtained by PCA of T-RFLP data indicated that bacterial population and diversity only in intestine change when *A. fulica* undergoes estivation. During the estivation state, the change in bacterial load and community structure might be due to passive change caused by the change of environment and nutritional conditions in the GI tract. Snails become least dependent on intestinal digestion and absorption. Because of this minimal dependency on intestinal function, the bacterial diversity and load only in intestine might be changing. Recently, the complexity of bacterial communities occurring in crop and intestine of the digestive tracts of field-collected *A. fulica* were assessed and then compared with those from groups of snails that were reared in the laboratory on a sugarcane-based diet (Cardoso et al. 2012). Our study is different from this report in having studied the bacterial community structures in different regions of the GI tract and then compared the bacterial load and community structure in different GI tract regions of active and estivating snails.

## Conclusion

Based on culture-independent and culture-dependent methods, this study for the first time demonstrates that bacterial flora in GI tract of *A. fulica* is complex and is dominated by members of *γ-Proteobacteria*, *Tenericutes*, and *Firmicutes*. Members of *Lactococcus* and *Kurthia* were present throughout the whole GI tract. Among the different GI tract regions, esophagus harbors highest and stomach harbors lowest diversity. This work presents basic information about the bacterial diversity in the GI tract of the Giant African Snail and suggested that bacterial load significantly reduces in the whole GI tract when snail enters estivation state. Our future work will include the study of diversity and characterization of metabolically active bacteria from different GI tract regions as well as isolation and characterization of lignocellulose degrading bacteria. Few of the bacterial isolates from this study will be further evaluated for their lignocellulose degrading potential.

## Supporting Information

**Figure S1.** Bayesian phylogenetic tree showing affiliations of 16S rRNA gene sequences from esophagus library. The tree shows relationship between 16S rRNA gene sequences recovered from clone libraries and reference sequences obtained from the NCBI database. One sequence per OTU is shown, and GenBank accession numbers of reference sequences are given in brackets. Bayesian posterior probabilities (based on the mean of three separate analyses) are shown. The scale bar represents 1% substitution per site. The aligned graph shows the number of sequences in each OTU.

**Figure S2.** Bayesian phylogenetic tree showing affiliations of 16S rRNA gene sequences from crop library. The tree shows relationship between 16S rRNA gene sequences recovered from clone libraries and reference sequences obtained from the NCBI database. One sequence per OTU is shown and GenBank accession numbers of reference sequences are given in brackets. Bayesian posterior probabilities (based on the mean of three separate analyses) are shown. The scale bar represents 1% substitution per site. The aligned graph shows the number of sequences in each OTU.

**Figure S3.** Bayesian phylogenetic tree showing affiliations of 16S rRNA gene sequences from stomach library. The tree shows relationship between 16S rRNA gene sequences recovered from clone libraries and reference sequences obtained from the NCBI database. One sequence per OTU is shown, and GenBank accession numbers of reference sequences are given in brackets. Bayesian posterior probabilities (based on the mean of three separate analyses) are shown. The scale bar represents 1% substitution per site. The aligned graph shows the number of sequences in each OTU.

**Figure S4.** Bayesian phylogenetic tree showing affiliations of 16S rRNA gene sequences from intestine library. The tree shows relationship between 16S rRNA gene sequences recovered from clone libraries and reference sequences obtained from the NCBI database. One sequence per OTU is shown, and GenBank accession numbers of reference sequences are given in brackets. Bayesian posterior probabilities (based on the mean of three separate analyses) are shown. The scale bar represents 1% substitution per site. The aligned graph shows the number of sequences in each OTU.

**Figure S5.** Bayesian phylogenetic tree showing affiliations of 16S rRNA gene sequences from rectum library. The tree shows relationship between 16S rRNA gene sequences recovered from clone libraries and reference sequences obtained from the NCBI database. One sequence per OTU is shown and GenBank accession numbers of reference sequences are given in brackets. Bayesian posterior probabilities (based on the mean of three separate analyses) are shown. The scale bar represents 1% substitution per site. The aligned graph shows the number of sequences in each OTU.

**Figure S6.** Venn diagram showing the total number of operational taxonomic units (OTUs) in each library and observed overlap of OTUs between different libraries.

**Figure S7.** A dendrogram from UniFrac environment cluster analysis of 16S rRNA gene clone libraries. Analysis involved weighted data; numbers at nodes indicate the frequency with which nodes were supported by environment cluster analysis.
