# Supplementary material for: Bacterial diversity in different regions of gastrointestinal tract of Giant African Snail (Achatina fulica)
Source: Microbiologyopen. 2012 Oct 19;1(4):415–26. doi: 10.1002/mbo3.38 (PMC3535387; doi:10.1002/mbo3.38)
Supplement: Supplementary file 1 [file mbo30001-0415-SD1.doc]

**Supplementary figures**


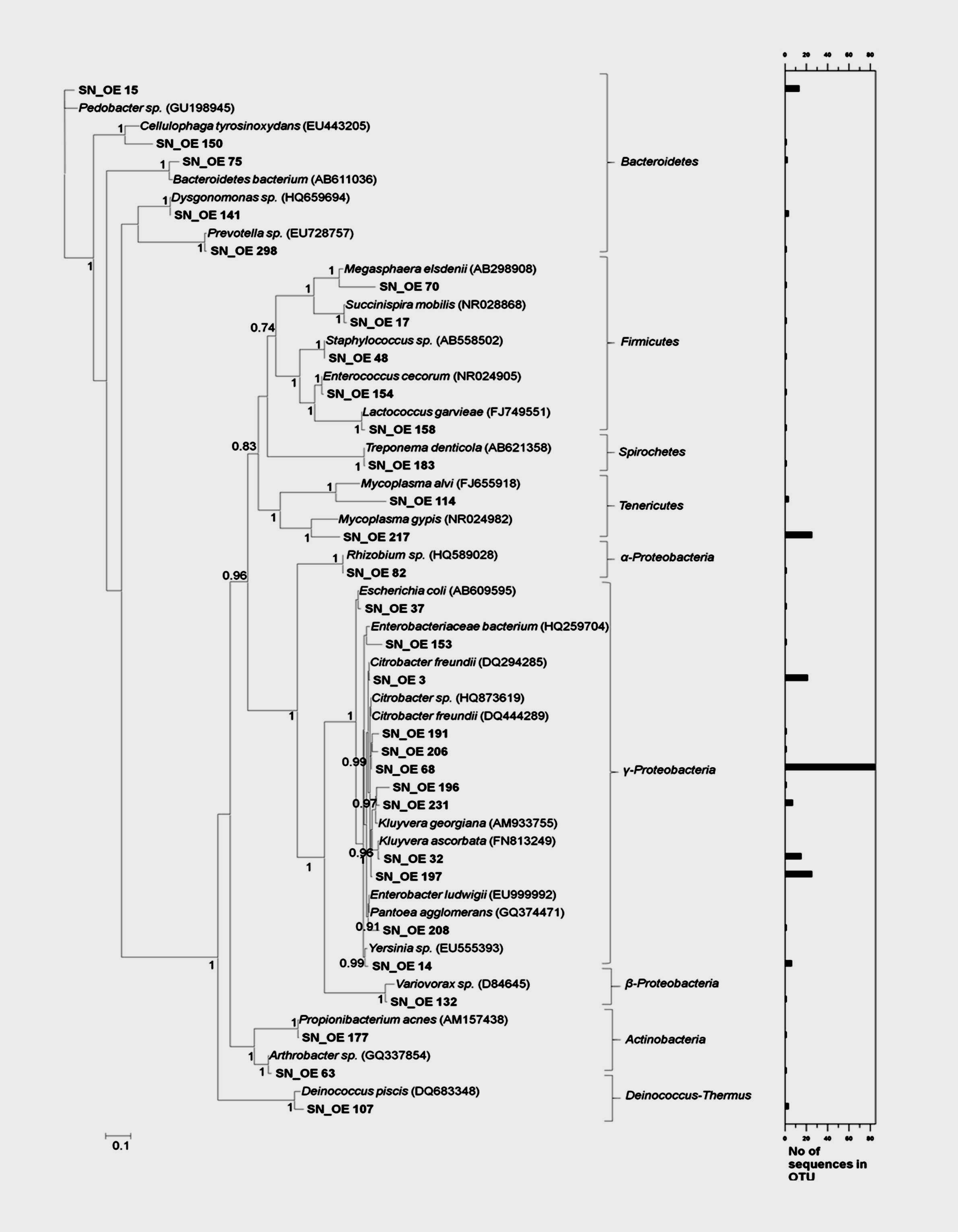


**Supplementary Figure 1**


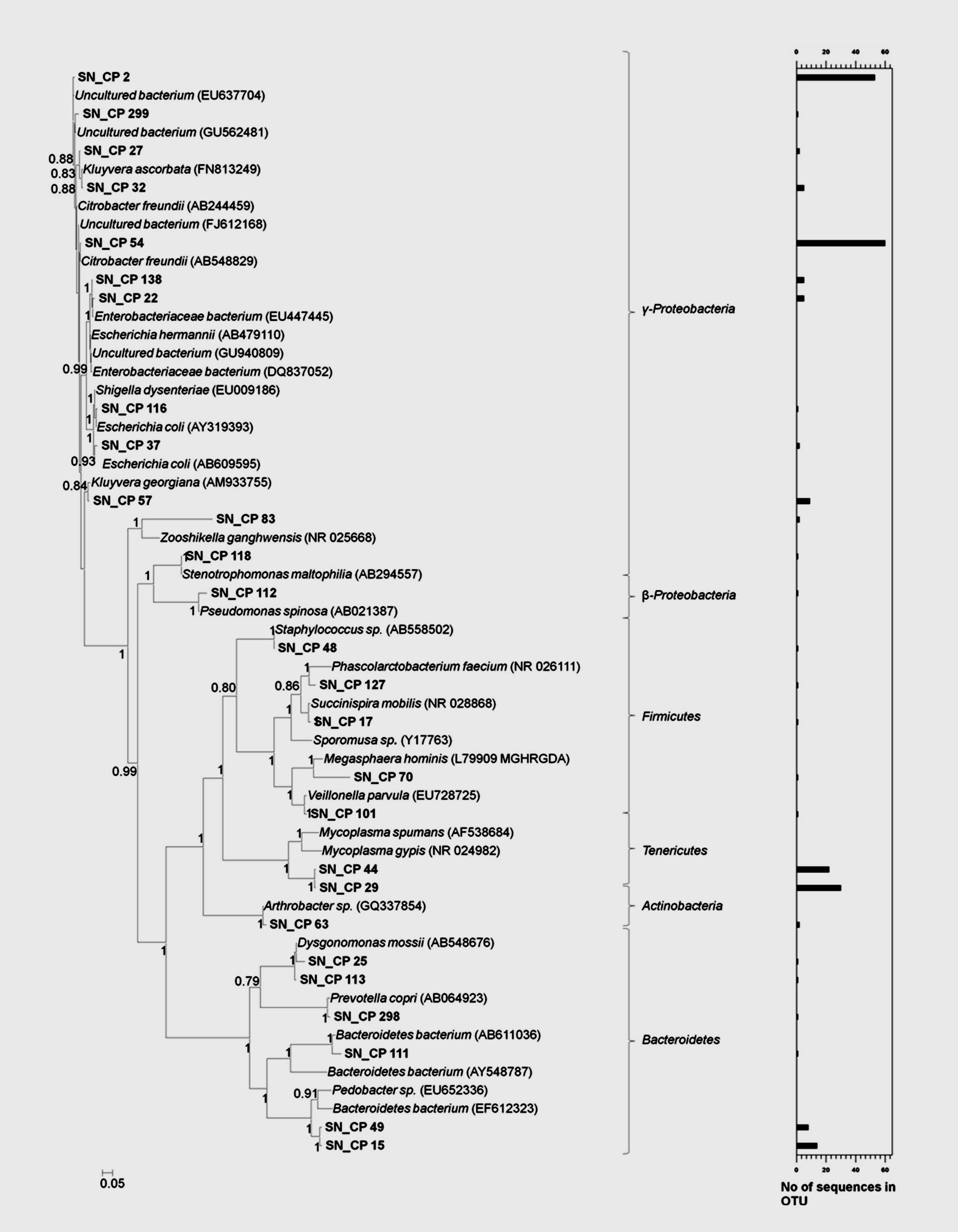


**Supplementary Figure 2**


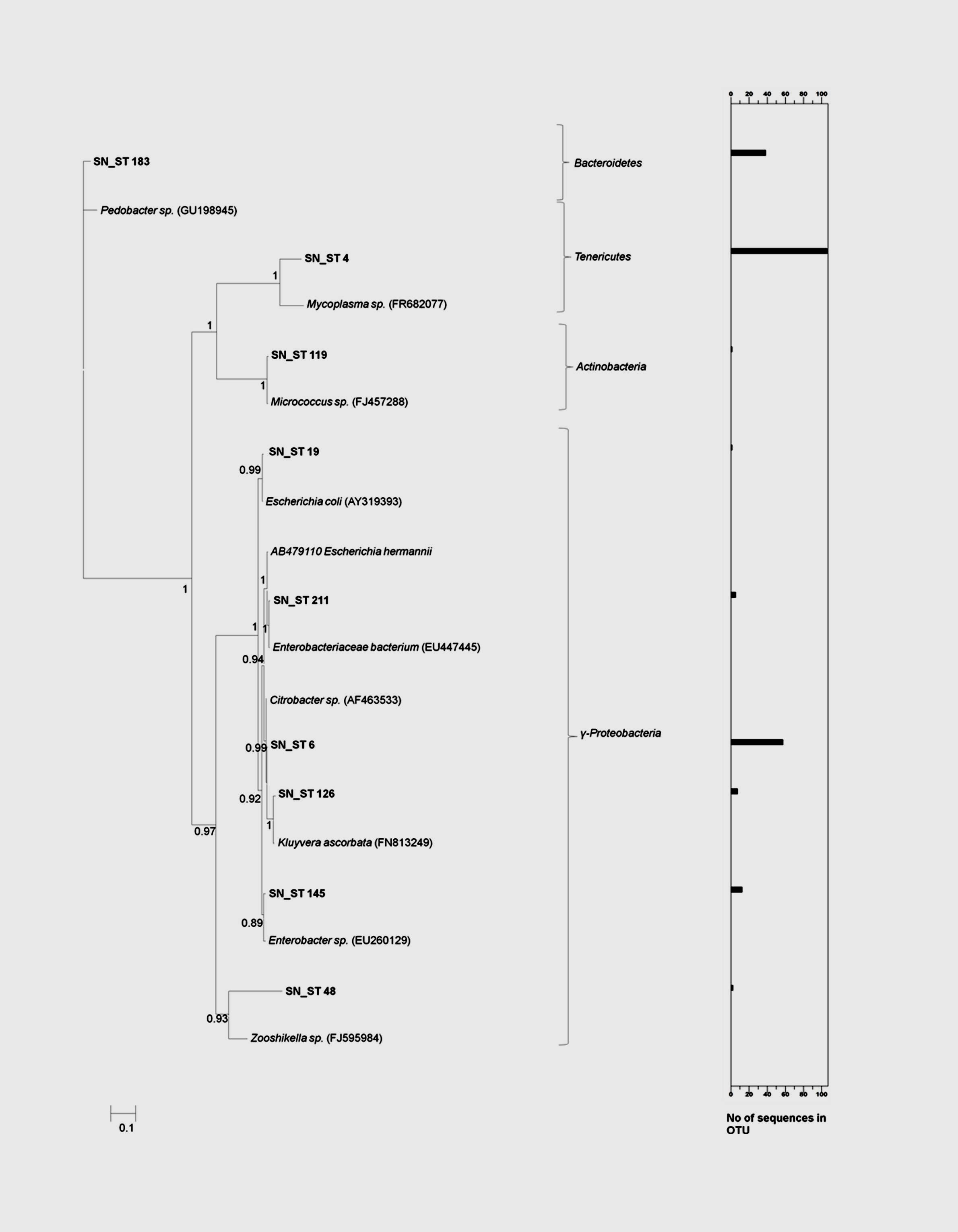


**Supplementary Figure 3**


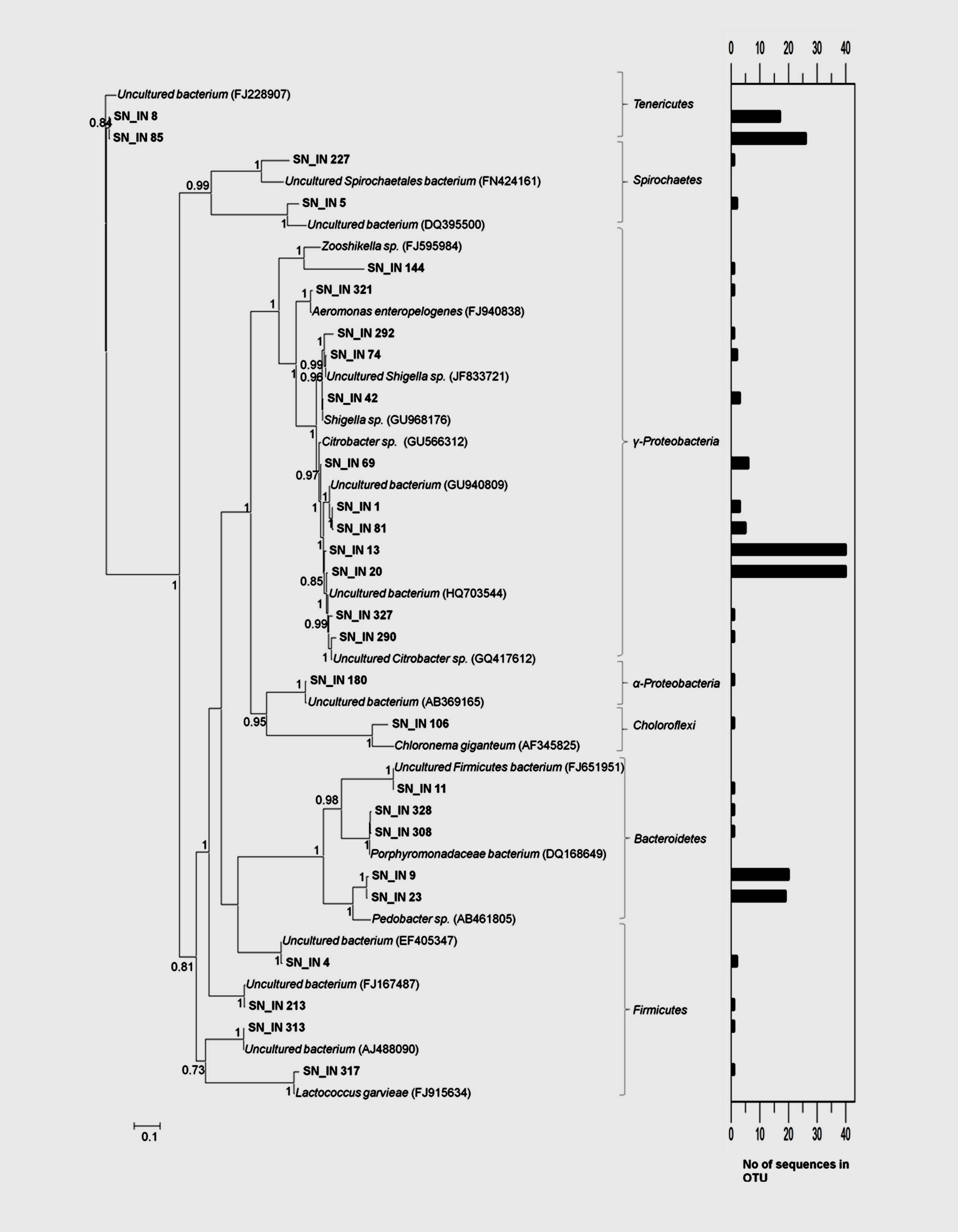


**Supplementary Figure 4**


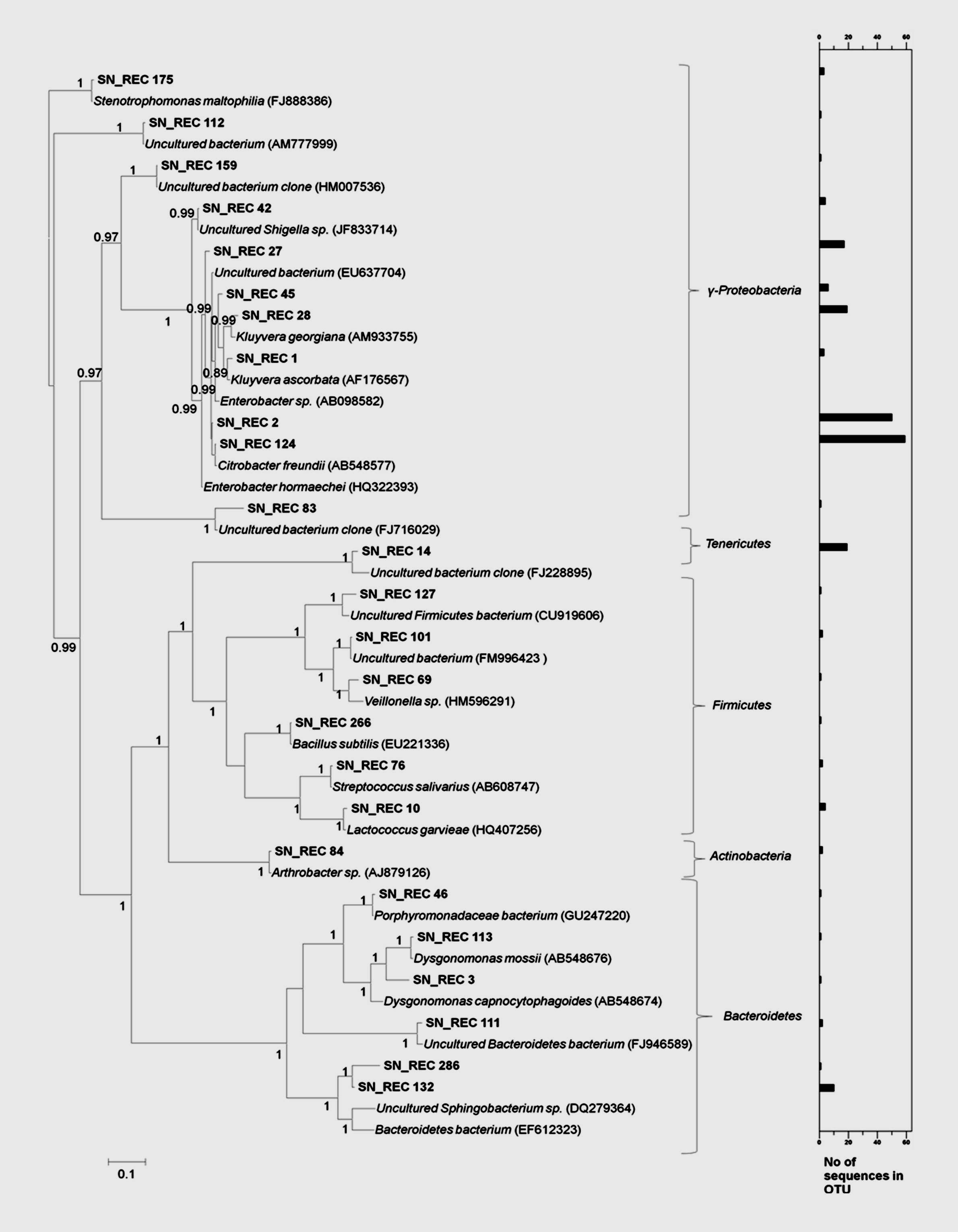


**Supplementary Figure 5**


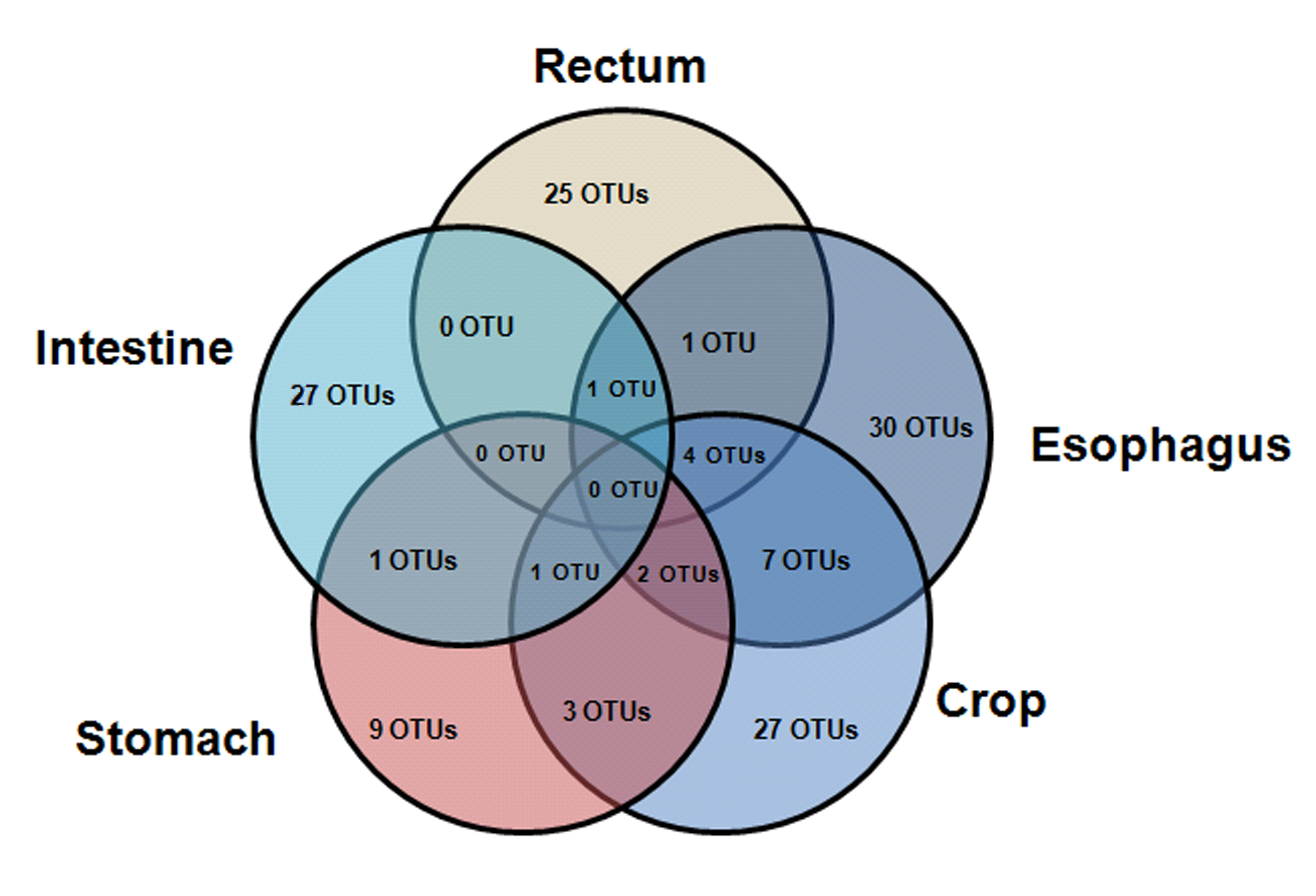


**Supplementary Figure 6**


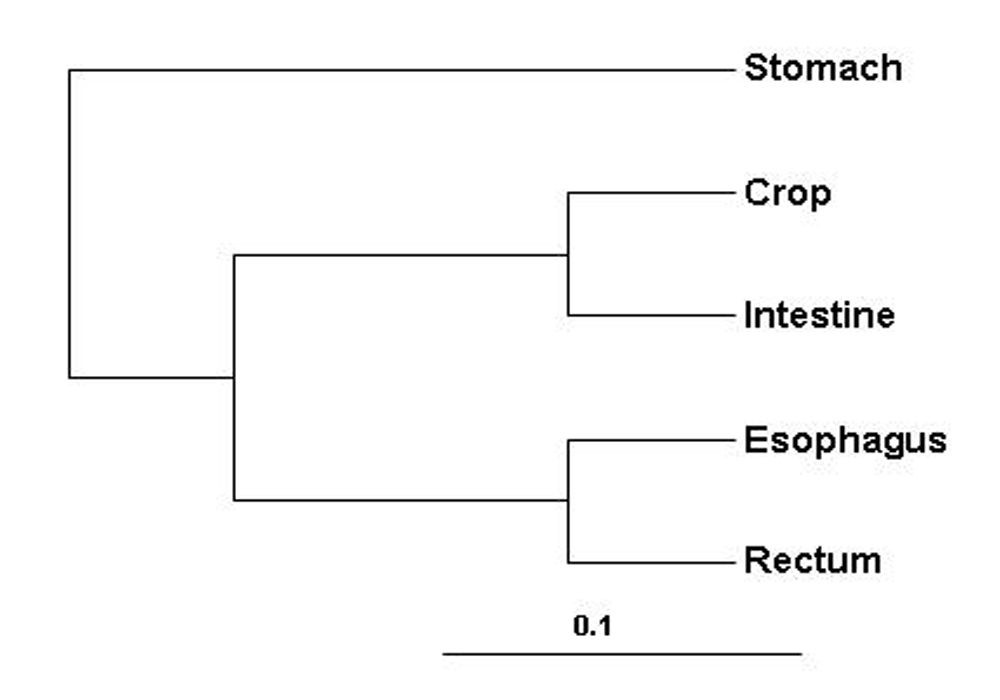


**Supplementary Figure 7**
